# Supplementary material for: Clinical and biomarker results from a phase II trial of combined cabozantinib and durvalumab in patients with chemotherapy-refractory colorectal cancer (CRC): CAMILLA CRC cohort
Source: Nat Commun. 2024 Feb 20;15:1533. doi: 10.1038/s41467-024-45960-2 (PMC10879200; doi:10.1038/s41467-024-45960-2)
Supplement: Supplementary file 1 — Supplementary Information [file 41467_2024_45960_MOESM1_ESM.pdf]

**Supplemental Figure 1. Workflow used for digital spatial profiling of clinical samples**. **A**) Pathologist review of an H&E-stained slides used to verify presence of sufficient tumor tissue in the biopsied material. **B**) An unstained serial section was then used for overnight hybridization using ISH probes conjugated to GeoMx DSP barcodes. Fluorescent antibodies against panCK (green) and CD45 (red) were used as morphological markers to identify epithelial cells and immune cells, respectively; Syto 13 dye (blue) was used to label nuclei. Regions of interest (ROIs) were selected using the morphology markers as guides. The ROIs were segmented into tumor cells and surrounding stromal cells including the immune cells followed by selective collection of their corresponding DSP barcodes by the GeoMx instrument. The DSP barcodes were quantified using next generation sequencing and the FASTQ files were processed to yield expression data for each mRNA target in the CTA panel.

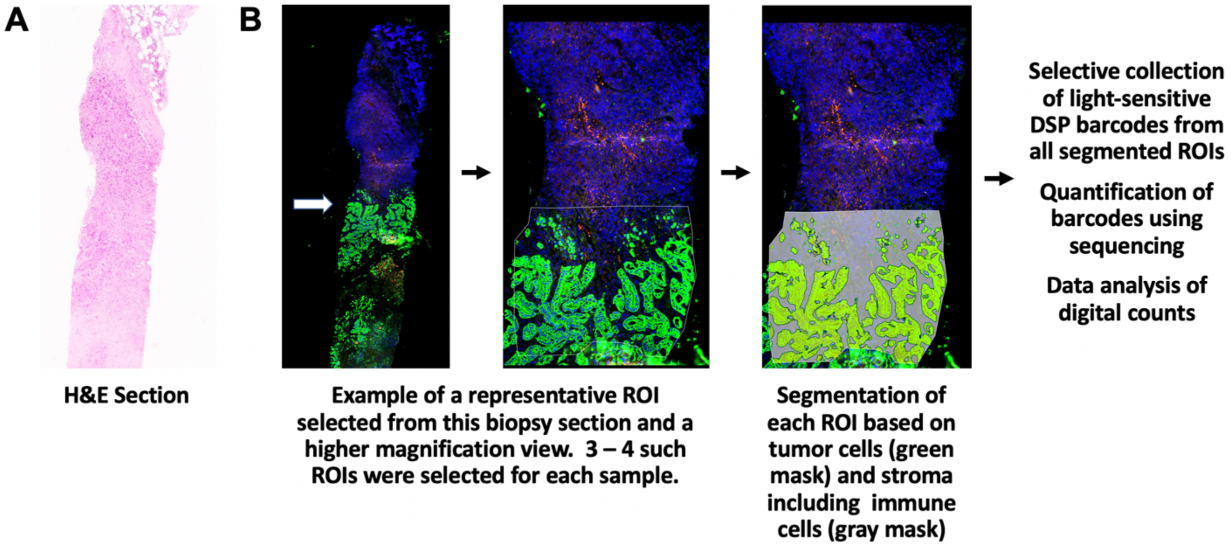

**Supplemental Figure 2. Kaplan-Meier analysis of progression-free survival by *RAS* status.** Blue line represents the overall population. Green (*RAS* wild type) and red (*RAS* mutant) broken lines represent stratification by *RAS* mutation status.

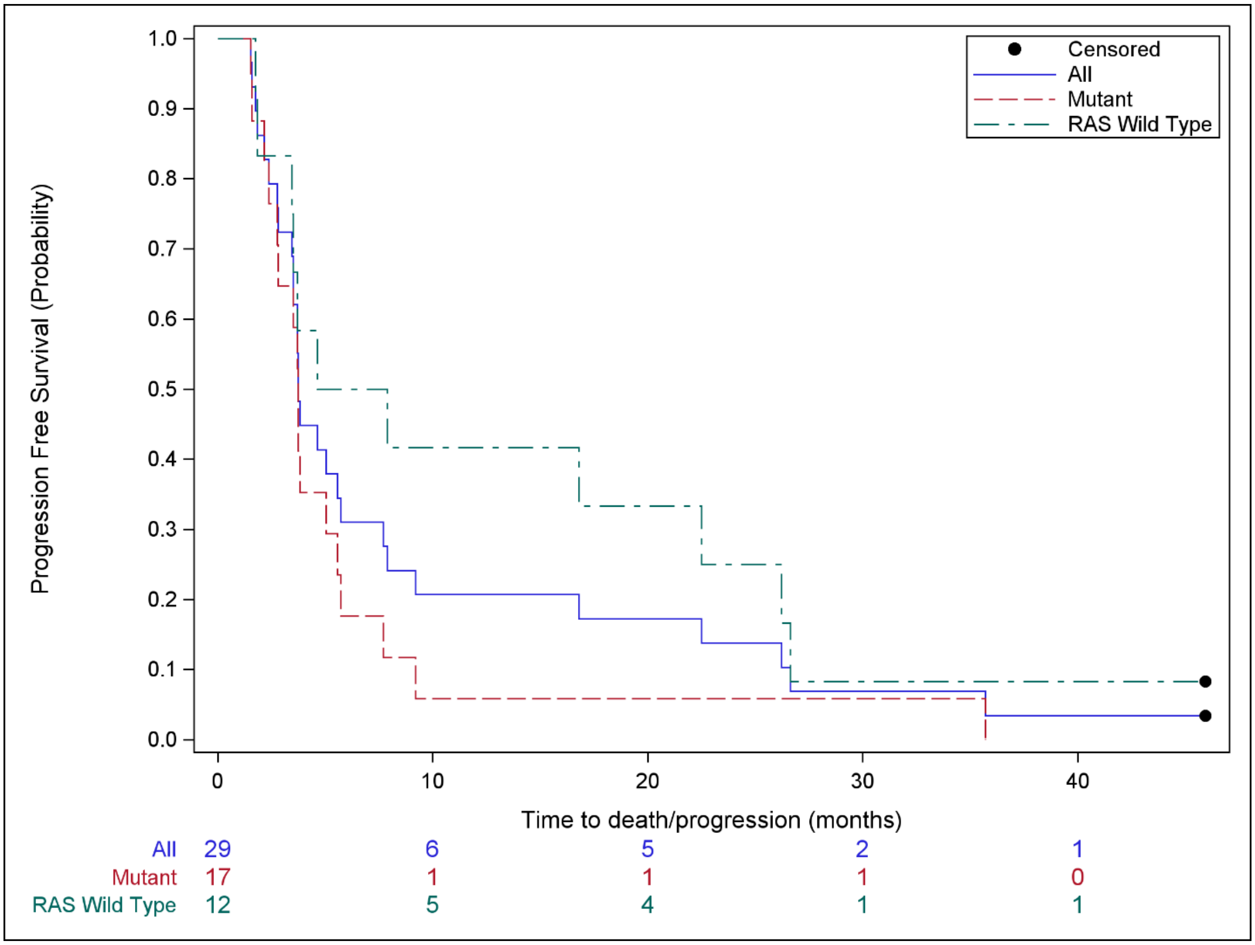

**Supplemental Figure 3. Kaplan-Meier analysis of overall survival by *RAS* status**. Blue line represents the overall population. Green (*RAS* wild type) and red (*RAS* mutant) broken lines represent stratification by *RAS* mutation status.

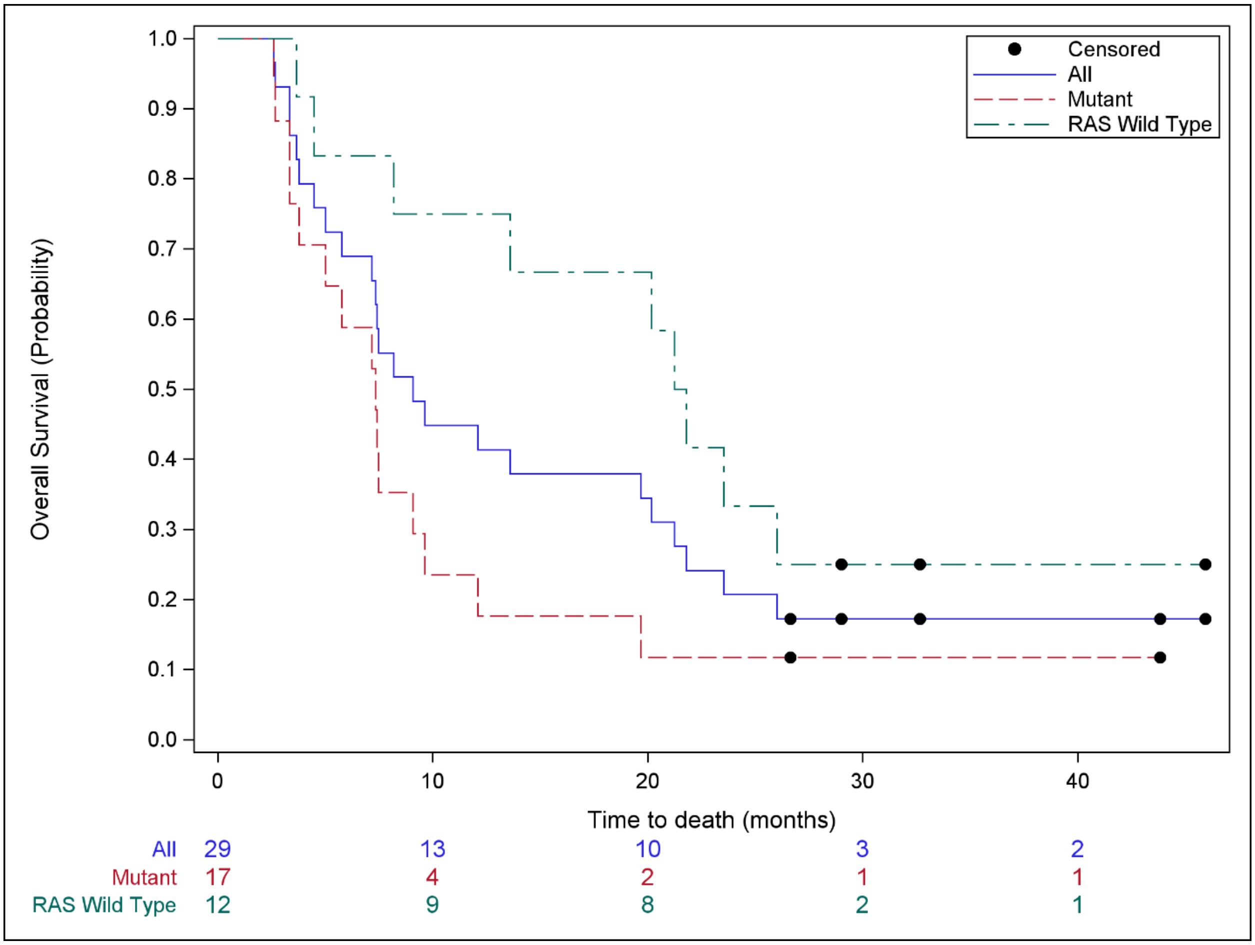

**Supplemental Figure 4. GeoMx spatial transcriptomic analysis results in *RAS* Wild Type (*RAS*-WT) subgroup. A)** Heatmap showing the scaled Q3-normalized expression of the differentially expressed genes (DEGs) between responders (R) and non-responders (NR) in tumor compartment in *RAS*-WT subgroup. Patients are depicted by key (ID). **B)** Volcano plot for the DEGs in R versus NR in tumor compartment. Significant DEGs are labeled and shown in red, with $log_2FC>1$ and adjusted p-value <0.05. NS, non-significant. **C)** Heatmap showing the scaled Q3-normalized expression of the DEGs between R and NR in stroma compartment in *RAS*-WT subgroup. Patients are depicted by key (ID). **D)** Volcano plot for the DEGs in R versus NR in stroma compartment in *RAS*-WT subgroup. Significant DEGs are labeled and shown in red, with $log_2FC>1$ and adjusted p-value <0.05. NS, non-significant.

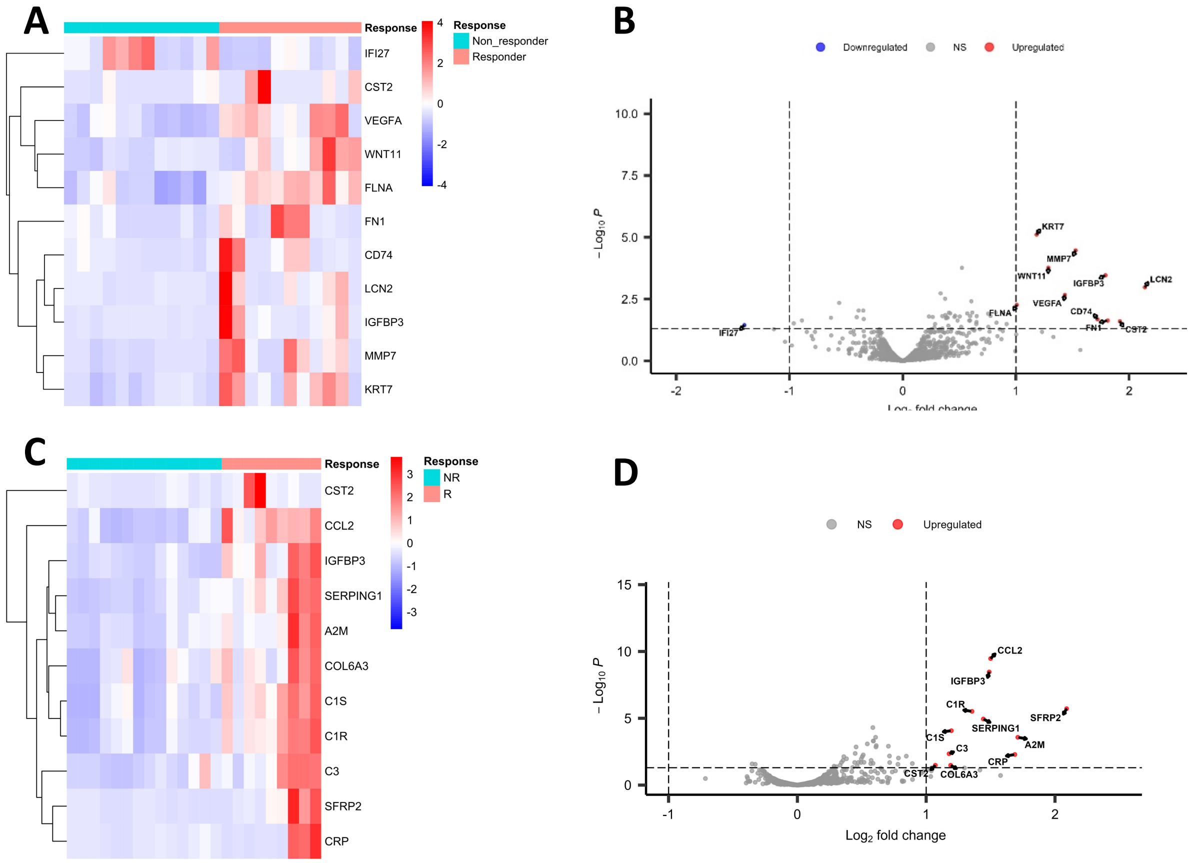

**Supplemental Figure 5. Gene ontology analysis results in *RAS*-WT subgroup. A)** Bar plots of selected significantly enriched gene ontology (GO) terms including enriched biological processes (BP), cellular components (CC) and molecular function (MF) in responders (R) versus non-responders (NR) in tumor compartment in *RAS*-WT subgroup. **B)** Bar plots of selected significant GO terms including BP, CC and MF, in R versus NR stroma compartment.

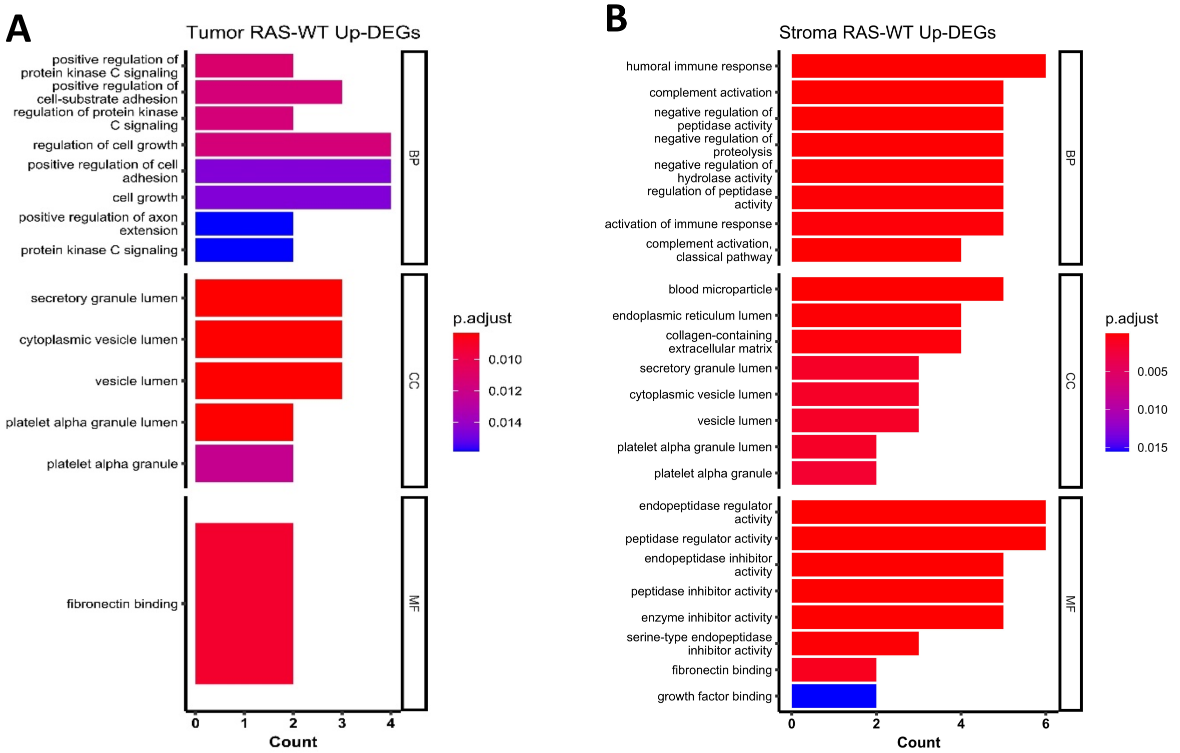

**Supplemental Figure 6. Gene Set Variation Analysis (GSVA) of significant differentially enriched pathways in responders versus non-responders in *RAS*-WT subgroup.** The y-axis represents annotated gene sets from the Nanostring Cancer Transcriptome Atlas. The pathways are organized within modules of Cell Function, Metabolism, Immune Response, Innate and Adaptive Immunity and Signaling Pathways. The x-axis represents the fold change difference of differentially enriched pathways in responders in comparison to non-responders in *RAS*-WT subgroup. Upregulated pathways are tinted in red and downregulated pathways are tinted in blue. **A)** GSVA of tumor epithelial compartment showing significant differentially enriched pathways in *RAS*-WT responders. **B)** GSVA of stroma compartment showing significant differentially enriched pathways in *RAS*-WT responders.

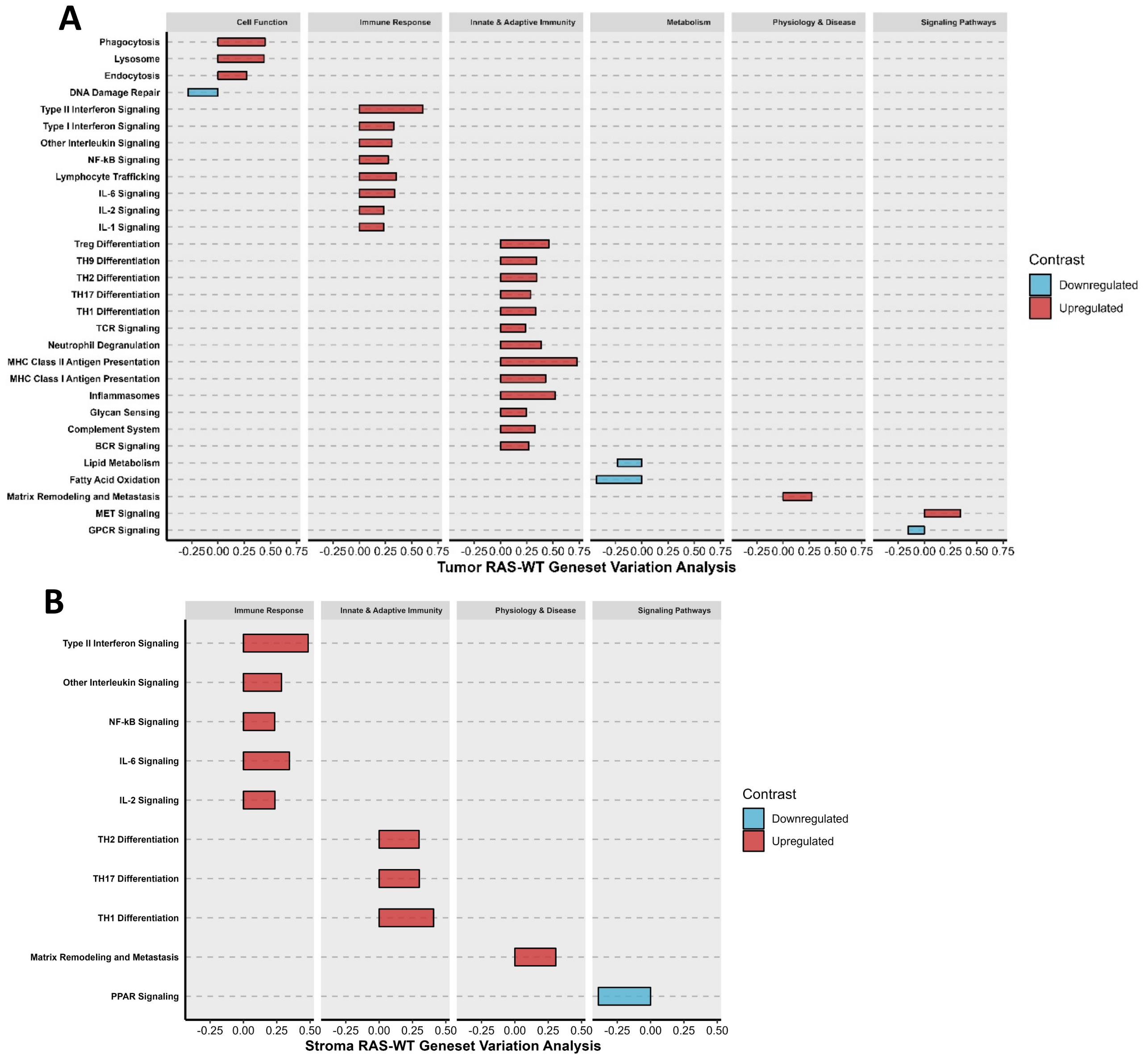

**Supplemental Figure 7. Cell type deconvolution by spatialDecon in *RAS*-WT subgroup. A)** Heatmap of scaled cell abundance scores (scaled Beta-values) in the tumor epithelial compartment in *RAS*-WT subgroup. Patients are depicted by key (ID). Plasmacytoid Dendritic cells (pDCs), myeloid Dendritic cells (mDCs), conventional monocytes (monocytes. C), non-conventional/intermediate monocytes (monocytes NC.I). **B)** Boxplots showing differences of cell infiltration between responders (R) and non-responders (NR) in tumor epithelial compartment in *RAS*-WT subgroup. Statistical significance was tested using Wilcoxon's rank sum test. **C)** Heatmap of scaled cell abundance scores (scaled Beta-values) in stroma compartment in *RAS*-WT subgroup. Patients are depicted by Key (ID). **D)** Boxplots showing differences of cell infiltration between R and NR in stroma compartment in *RAS*-WT subgroup. Statistical significance was tested using Wilcoxon's rank sum test.

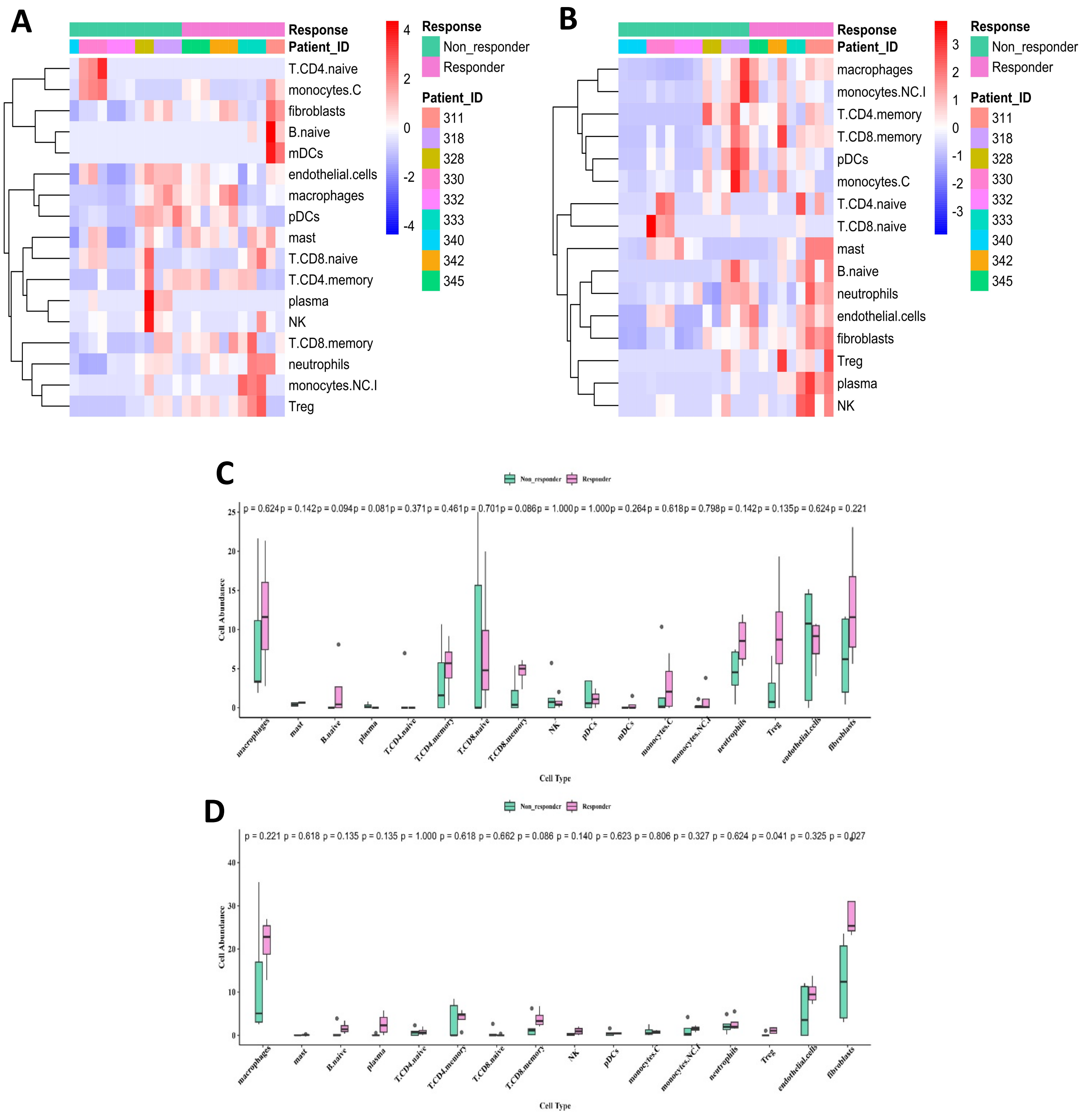

**Supplemental Figure 8. T-cell inflamed gene expression signature (TIS) in *RAS*-WT subgroup. A)** Heatmap of genes in T-cell inflamed gene expression signature representing the scaled expression of TIS genes. Patients are depicted by key (ID). **B)** Boxplots showing differences in the TIS scores between responders (R) and non-responders (NR) in *RAS*-WT subgroup. The y-axis represents the TIS score. Statistical significance was tested using Wilcoxon's rank sum test. Responders (R), non-responders (NR).

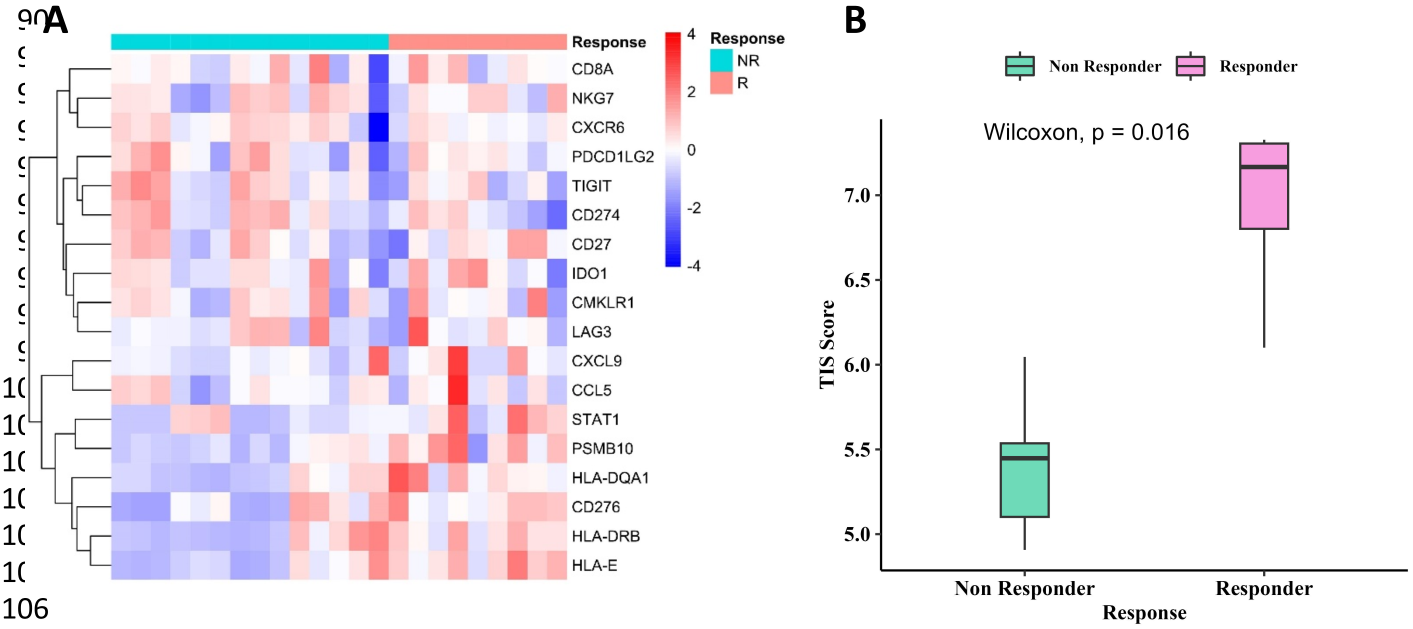

**Supplemental Figure 9. Consort Diagram.** Study Consort Diagram.

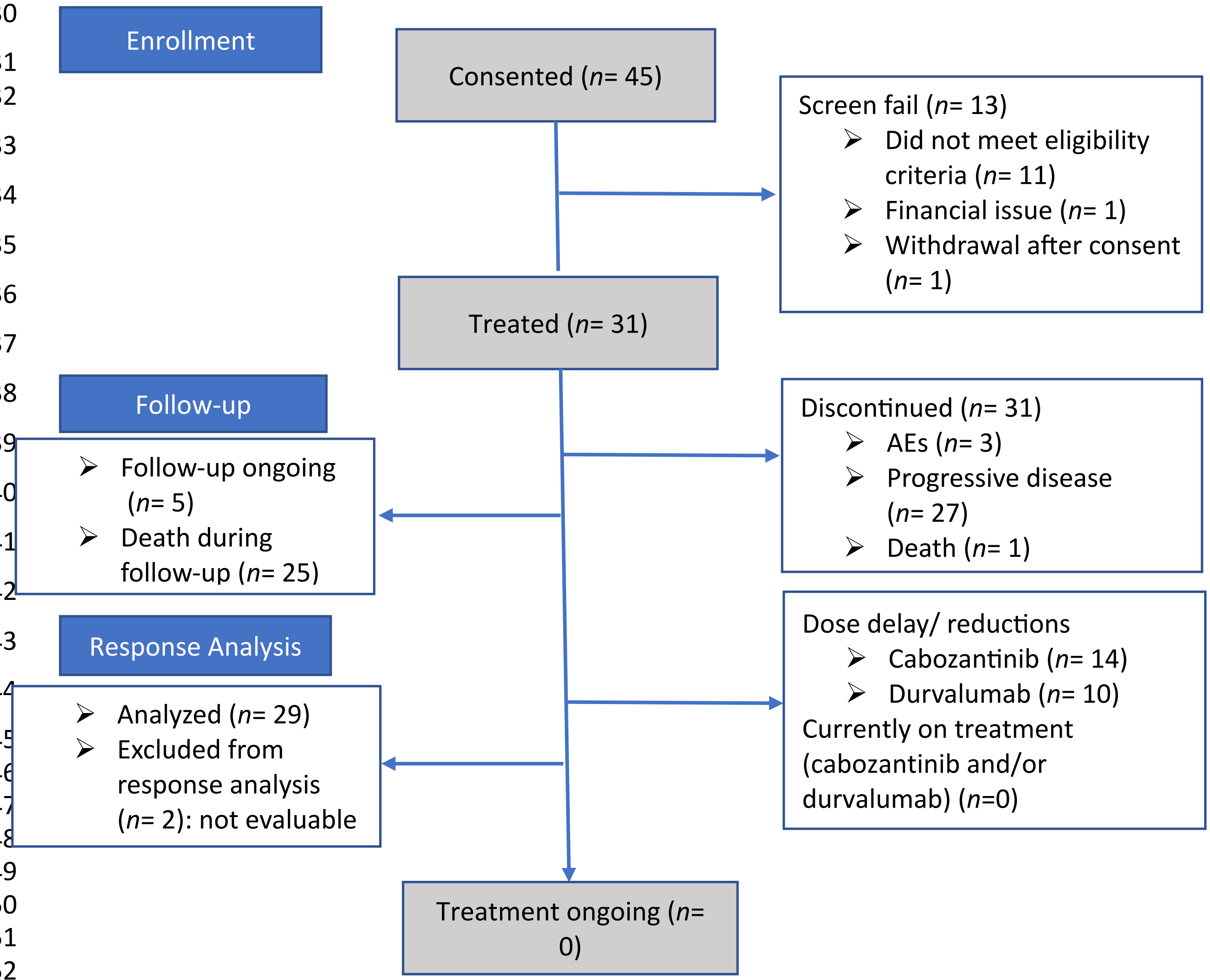

**Supplemental Figure 10. Spider plot of individual patient responses**. Colors of lines represent best overall response (green, partial response; blue, stable disease; red, progressive disease). A star denotes a *RAS* wild type patient and # represents a patient with no liver metastasis.

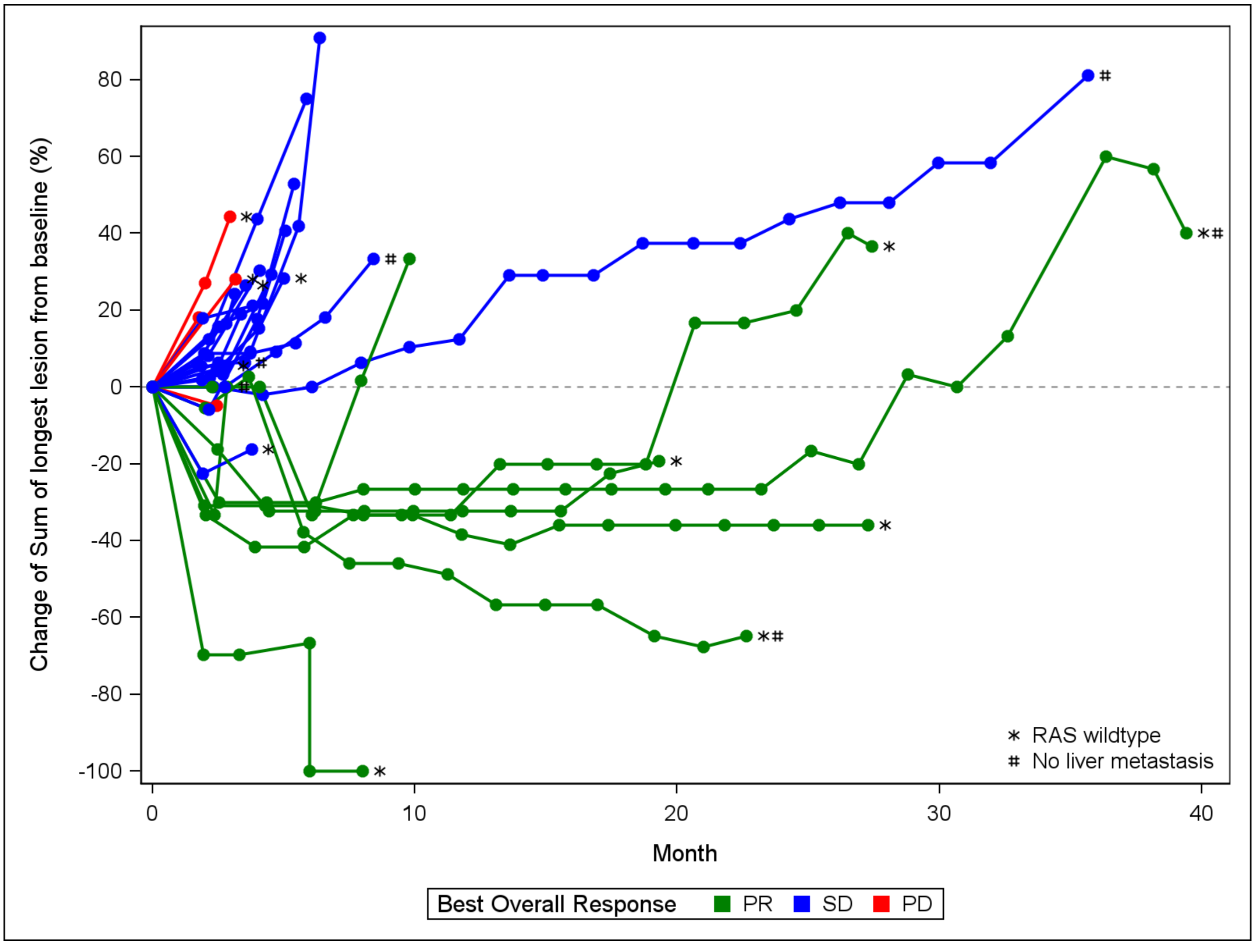

**Supplemental Figure 11. Kaplan-Meier analysis of progression-free survival**. X axis depicts time to death or progression in months. Y axis depicts the probability of progression free survival.

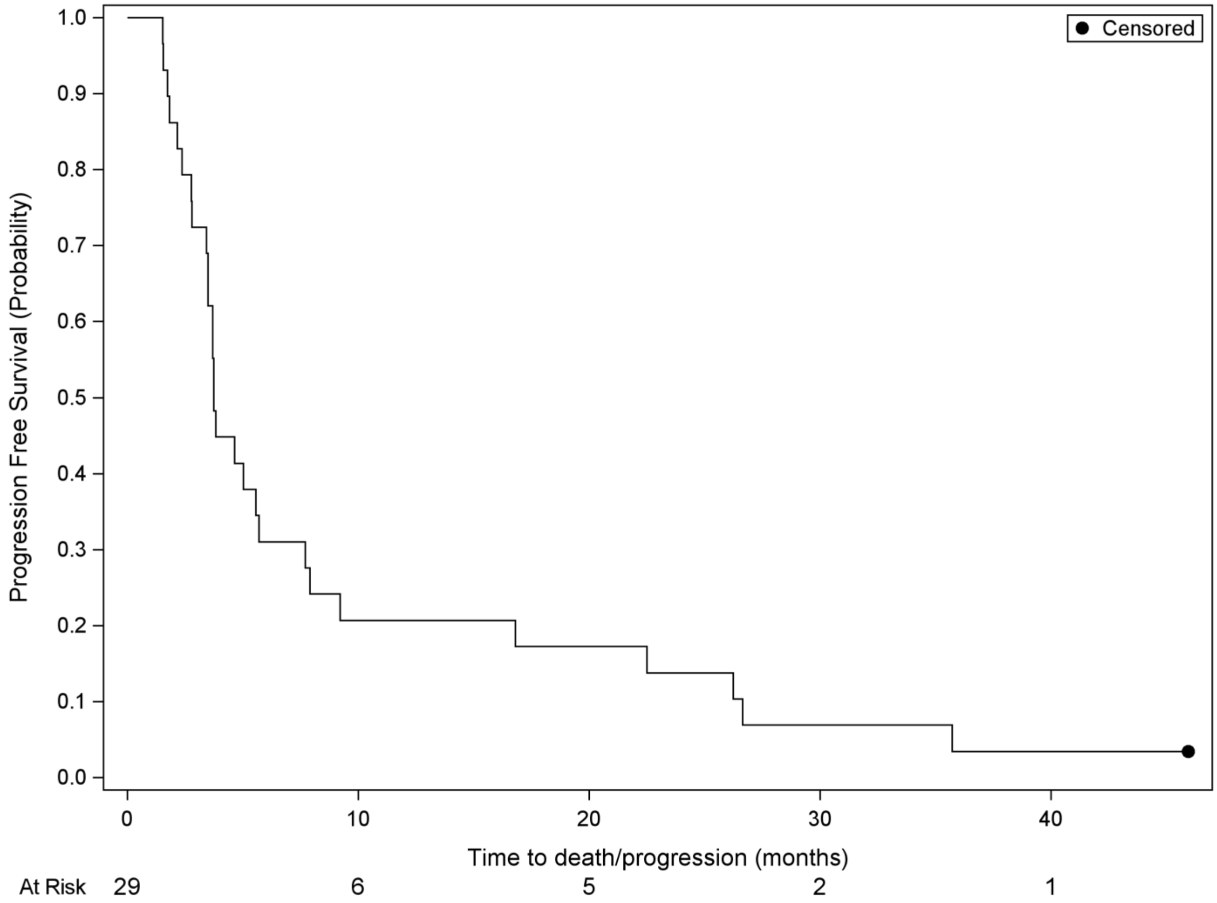

**Supplemental Figure 12. Kaplan-Meier analysis of overall survival**. X axis depicts time to death in months. Y axis depicts the probability of overall survival.

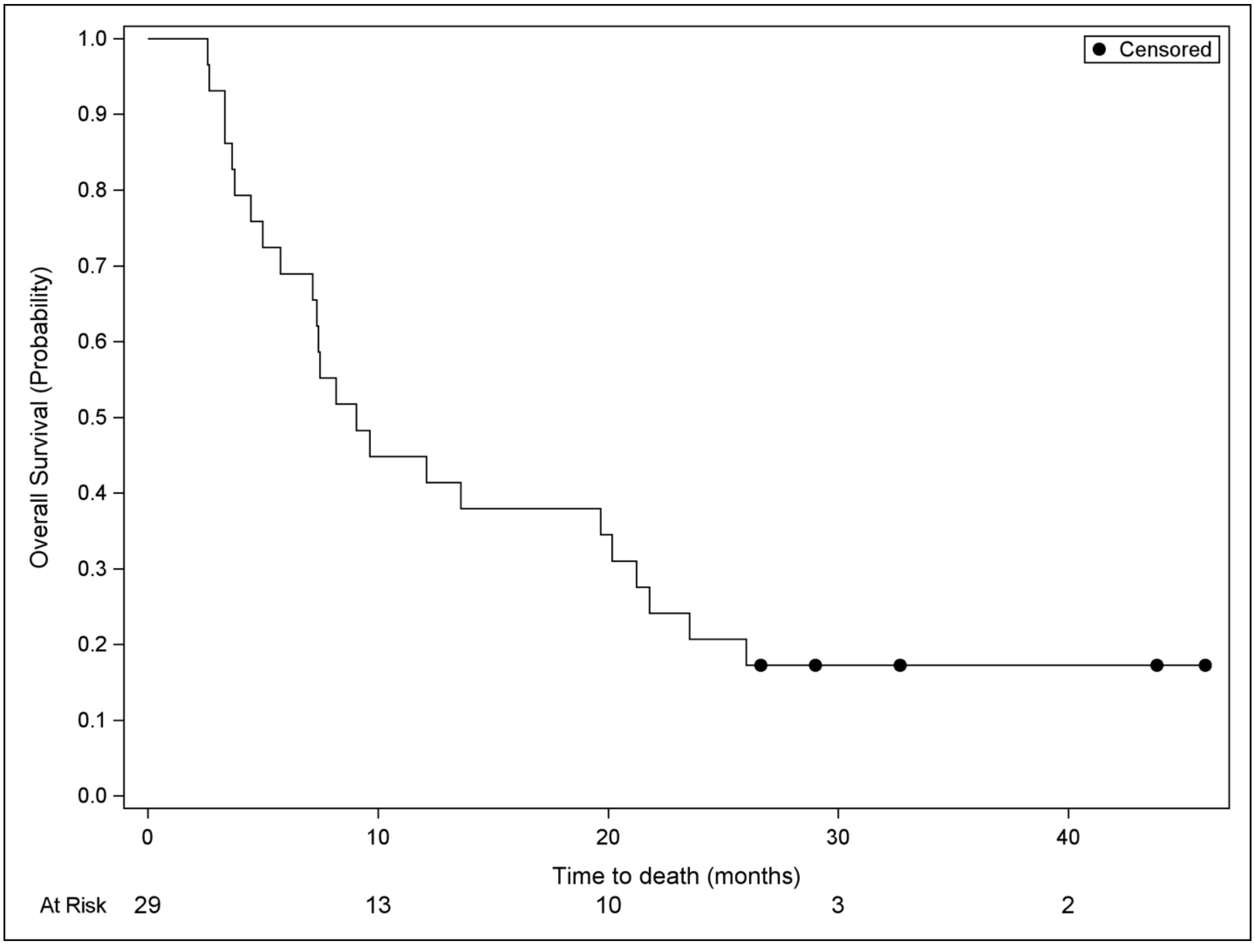


**Supplemental Table S1. *RAS* wild type subgroup summary of efficacy data.**

| Variable | *RAS* Wild Type (N=12) |
|---|---|
| Overall objective responses (ORR, 95% CI) | 6 (50%, 21.1-78.9) |
| Confirmed partial response (ORR, 95% CI) | 6 (50%, 21.1-78.9) |
| Best overall response | |
| Complete response | 0 |
| Partial response | 6 (50 %) |
| Stable disease | 4 (33.3%) |
| Progressive disease | 2 (16.6%) |
| Disease control rate | 10/12 (83.3%, 51.6-97.9) |

| | |
|---|---|
| Median progression-free survival | 6.3 months (1.8-26.2 months) |
| Median overall survival | 21.5 months(4.5-not estimable) |
| 4-month PFS rate | n/a |
| 6-month PFS rate | n/a |

Note. Data area presented as No. (%, lower and upper limits of 95% confidence interval) unless otherwise noted.

**Supplemental Table S2. Clinical and molecular baseline characteristics and tumor biopsy sites for patient samples tested in the DSP study.**

| Study ID | Gender | Age | Primary colon tumor Side | *RAS* gene status | Number of Prior Lines of therapy | Prior EGFR inhibitor | Number of Metastatic Sites <3 / ≥3 | Liver metastasis | Best response | Progression free time (in days) | Survival time (in days) | Biopsy Site | Response |
|---|---|---|---|---|---|---|---|---|---|---|---|---|---|
| 311 | F | 76 | Right | Wild type | 3 | yes | > 3 | no | PR | 1149 | 1149 | lung | Responder |
| 312 | M | 53 | Left/ rectum | *KRAS* mutant | 3 | no | > 3 | yes | SD | 111 | 215 | liver | Non responder |
| 314 | M | 66 | Left | *KRAS* mutant | 2 | no | > 3 | yes | SD | 112 | 222 | liver | Non responder |
| 315 | M | 62 | Left | *KRAS* mutant | 2 | no | > 3 | yes | SD | 105 | 272 | liver | Non responder |
| 316 | F | 57 | Left | *KRAS* mutant | 2 | no | > 3 | no | SD | 231 | 590 | lung | Non responder |
| 318 | F | 51 | Left/ rectum | Wild type | 3 | yes | > 3 | yes | SD | 139 | 654 | liver | Non responder |
| 321 | F | 51 | Right | *KRAS* mutant | 2 | no | > 3 | yes | PD | 47 | 80 | lung | Non responder |
| 327 | F | 52 | Left/ rectum | *NRAS* mutant | 3 | yes | > 3 | yes | SD | 112 | 224 | liver | Non responder |
| 328 | M | 27 | Left/ rectum | Wild type | 2 | yes | > 3 | yes | SD | 105 | 245 | liver | Non responder |
| 330 | F | 61 | Left | Wild type | 3 | yes | > 3 | yes | PD | 55 | 605 | adnexal mass | Non responder |
| 332 | F | 36 | Left | Wild type | 3 | no | > 3 | yes | SD | 103 | 110 | liver | Non responder |
| 333 | F | 64 | Left/ rectum | Wild type | 3 | yes | > 3 | no | PR | 675 | 704 | retroperitoneal mass | Responder |
| 338 | F | 50 | Left/ rectum | *KRAS* mutant | 3 | no | > 3 | no | SD | 83 | 113 | lymph node | Non responder |
| 340 | F | 57 | Left | Wild type | 3 | yes | > 3 | yes | PD | 52 | 134 | liver | Non responder |
| 341 | M | 51 | Left/ rectum | *KRAS* mutant | 2 | no | > 3 | yes | SD | 46 | 100 | liver | Non responder |
| 342 | F | 70 | Left/ rectum | Wild type | 2 | yes | > 3 | yes | PR | 612 | 641 | liver | Responder |
| 343 | M | 62 | Left/ rectum | *NRAS* mutant | 2 | yes | > 3 | yes | SD | 171 | 173 | liver | Non responder |
| 344 | F | 70 | Left | *KRAS* mutant | 2 | no | > 3 | yes | SD | 84 | 150 | liver | Non responder |
| 345 | M | 65 | Left/ rectum | Wild type | 3 | yes | > 3 | yes | PR | 504 | 619 | liver | Responder |
| 353 | M | 63 | Left/ rectum | *KRAS* mutant | 2 | no | > 3 | yes | SD | 167 | 220 | liver | Non responder |

**Supplemental Table S3. Characteristics of patients with confirmed partial response to the investigational regimen.**

| | Tumor location | Type of Overall Tumor Response | Progression free time (months) | Presence of Liver Metastasis | Best response in liver metastatic lesions | *RAS* mutational status | Prior EGFR targeted therapy |
|---|---|---|---|---|---|---|---|
| Patient #1 | Right sided (Cecum) | PR | 38 | No | N/A | Wild type | Yes |
| Patient #2 | Left sided (Rectum) | PR | 25 | Yes | SD | Wild type | Yes |
| Patient #3 | Left sided (Rectum) | PR | 22 | No | N/A | Wild type | Yes |
| Patient #4 | Left sided (sigmoid) | PR | 8 | Yes | SD | Wild type | Yes |

| | | | | | | | |
|---|---|---|---|---|---|---|---|
| Patient #5 | Left sided (Rectum) | PR | 20 | Yes | PR | Wild type | Yes |
| Patient #6 | Left sided (Rectum) | PR | 17 | Yes | SD | Wild type | Yes |
