# Supplementary material for: Clinical and biomarker results from a phase II trial of combined cabozantinib and durvalumab in patients with chemotherapy-refractory colorectal cancer (CRC): CAMILLA CRC cohort
Source: Nat Commun. 2024 Feb 20;15:1533. doi: 10.1038/s41467-024-45960-2 (PMC10879200; doi:10.1038/s41467-024-45960-2)
Supplement: Supplementary file 3 — Description of additional supplementary files [file 41467_2024_45960_MOESM3_ESM.pdf]

DESCRIPTION OF ADDITIONAL SUPPLEMENTARY FILES DOCUMENT

Title: Supplementary Data 1

Description: The list of the differen4ally expressed genes (DEGs) defined as genes with |log2 FoldChange| >=1 and BH-adjusted P-value<0.05 in tumor and stromal compartments between responders vs. non responders based on the linear model. Genes with Log2FC>1 were considered upregulated, and genes with Log2FC<-1 were considered downregulated. Enriched Gene Ontology (GO) terms for the upregulated DEGs in the tumor and stromal compartments defined with BH-adjusted p-values<0.05 using overrepresenta4on analysis from clusterProfiler package.

Title: Supplementary Data 2

Description: The list of Gene Set Varia4on Analysis (GSVA) results for the Nanostring gene sets in tumor and stromal compartments between responders vs. non-responders. Significantly enriched gene sets were defined with BH-corrected p-values<0.05 from the limma linear model.

Title: Supplementary Data 3

Description: The list of Gene Set Varia4on Analysis (GSVA) results for the Reactome curated gene sets in tumor and stromal compartments between responders vs. non-responders. Significantly enriched gene sets were defined with BH-corrected p-values<0.05 from the limma linear model. Pathways with Log2FC>0 were considered upregulated, and pathways with Log2FC<0 were considered downregulated.

Title: Supplementary Data 4

Description: Comparison of the cell abundance es4mates between responders and nonresponders in tumor and stromal compartment. The LASSO coefficients for the T-cell inflamed gene signature using the penalized regression model in the stromal compartment.

Title: Supplementary Data 5

Description: The list of the differen4ally expressed genes (DEGs) defined as genes with |log2 FoldChange| >=1 and BH-adjusted P-value<0.05 in tumor and stromal compartments between responders vs. non responders in RAS-WT pa4ents based on the linear model. Genes with Log2FC>1 were considered upregulated, and genes with Log2FC<-1 were considered downregulated.

Title: Supplementary Data 6

Description: Enriched Gene Ontology (GO) terms for the upregulated DEGs in the RAS-WT tumor compartment defined with BH-adjusted p-values<0.05 using overrepresenta4on analysis from clusterProfiler package. The list of Gene Set Varia4on Analysis (GSVA) results for the Nanostring gene sets in RAS-WT tumor and stromal compartments between responders vs. non-responders. Significantly enriched gene sets were defined with BH-corrected p-values<0.05 from the limma linear model.

Title: Supplementary Data 7

Description: Most common treatment-related adverse events (TRAEs) by preferred term and grade. Most common TRAEs by preferred term and grade.
