# Supplementary material for: Clinical and biomarker results from a phase II trial of combined cabozantinib and durvalumab in patients with chemotherapy-refractory colorectal cancer (CRC): CAMILLA CRC cohort
Source: Nat Commun. 2024 Feb 20;15:1533. doi: 10.1038/s41467-024-45960-2 (PMC10879200; doi:10.1038/s41467-024-45960-2)
Supplement: Supplementary file 10 — Supplementary Data 7 [file 41467_2024_45960_MOESM10_ESM.docx]

| **Preferred term, n (%)** | **Cabozantinib + Durvalumab (n=31)** | | | | | | |
| --- | --- | --- | --- | --- | --- | --- | --- |
|  | **< Grade 3** | **G1** | **G2** | **Grade ≥ 3** | **G3** | **G4** | **G5** |
| Fatigue | 20 (65%) | 9 | 11 | 2 (6%) | 2 | 0 | 0 |
| Transaminitis | 18 (58%) | 14 | 4 | 4 (13%) | 4 | 0 | 0 |
| Nausea | 18 (58%) | 16 | 2 | 0 (0%) | 0 | 0 | 0 |
| Hyperthyroidism | 15 (48%) | 14 | 1 | 1 (3%) | 1 | 0 | 0 |
| Diarrhea | 13 (42%) | 10 | 3 | 0 (0%) | 0 | 0 | 0 |
| Anorexia | 13 (42%) | 8 | 5 | 0 (0%) | 0 | 0 | 0 |
| Palmar-plantar erythrodysesthesia syndrome | 11 (35%) | 4 | 7 | 1 (3%) | 1 | 0 | 0 |
| Rash maculo-papular | 9 (29%) | 5 | 4 | 0 (0%) | 0 | 0 | 0 |
| Hypothyroidism | 7 (23%) | 6 | 1 | 0 (0%) | 0 | 0 | 0 |
| Headache | 7 (23%) | 7 | 0 | 0 (0%) | 0 | 0 | 0 |
| Hypertension | 6 (19%) | 2 | 4 | 1 (3%) | 1 | 0 | 0 |
| Mucositis oral | 6 (19%) | 5 | 1 | 1 (3%) | 1 | 0 | 0 |
| Dry skin | 6 (19%) | 6 | 0 | 0 (0%) | 0 | 0 | 0 |
| Hypokalemia | 6 (19%) | 6 | 0 | 0 (0%) | 0 | 0 | 0 |
| Weight loss | 5 (16%) | 1 | 4 | 0 (0%) | 0 | 0 | 0 |
| Edema limbs | 5 (16%) | 3 | 2 | 0 (0%) | 0 | 0 | 0 |
| Enterocolitis | 5 (16%) | 2 | 3 | 0 (0%) | 0 | 0 | 0 |
| Memory impairment | 4 (13%) | 3 | 1 | 0 (0%) | 0 | 0 | 0 |
| Proteinuria | 4 (13%) | 0 | 4 | 2 (6%) | 2 | 0 | 0 |
| Blood lactate dehydrogenase increased | 4 (13%) | 4 | 0 | 0 (0%) | 0 | 0 | 0 |
| Oral pain | 4 (13%) | 4 | 0 | 0 (0%) | 0 | 0 | 0 |
| Myalgia | 4 (13%) | 4 | 0 | 0 (0%) | 0 | 0 | 0 |
| Platelet count decreased | 3 (10%) | 1 | 2 | 1 (3%) | 1 | 0 | 0 |
| Blood bilirubin increased | 3 (10%) | 3 | 0 | 1 (3%) | 1 | 0 | 0 |
| Hypomagnesemia | 3 (10%) | 2 | 1 | 0 (0%) | 0 | 0 | 0 |
| Pulmonary hypertension | 3 (10%) | 2 | 1 | 0 (0%) | 0 | 0 | 0 |
| Hair color changes | 3 (10%) | 3 | 0 | 0 (0%) | 0 | 0 | 0 |
| Vomiting | 3 (10%) | 3 | 0 | 0 (0%) | 0 | 0 | 0 |
| Bloating | 3 (10%) | 3 | 0 | 0 (0%) | 0 | 0 | 0 |
| Alkaline phosphatase increased | 2 (6%) | 0 | 2 | 1 (3%) | 1 | 0 | 0 |
| Hypoalbuminemia | 2 (6%) | 0 | 2 | 0 (0%) | 0 | 0 | 0 |
| Thromboembolic event | 2 (6%) | 1 | 1 | 1 (3%) | 1 | 0 | 0 |
| Amnesia | 2 (6%) | 1 | 1 | 0 (0%) | 0 | 0 | 0 |
| Cough | 2 (6%) | 1 | 1 | 0 (0%) | 0 | 0 | 0 |
| Dyspnea | 2 (6%) | 1 | 1 | 0 (0%) | 0 | 0 | 0 |
| Abdominal pain | 2 (6%) | 2 | 0 | 0 (0%) | 0 | 0 | 0 |
| Agitation | 2 (6%) | 2 | 0 | 0 (0%) | 0 | 0 | 0 |
| Dry mouth | 2 (6%) | 2 | 0 | 0 (0%) | 0 | 0 | 0 |
| Hematochezia | 2 (6%) | 2 | 0 | 0 (0%) | 0 | 0 | 0 |
| Generalized muscle weakness | 2 (6%) | 2 | 0 | 0 (0%) | 0 | 0 | 0 |
| Rectal hemorrhage | 2 (6%) | 2 | 0 | 0 (0%) | 0 | 0 | 0 |
| Lipase increased | 1 (3%) | 0 | 1 | 1 (3%) | 0 | 1 | 0 |
| White blood cell decreased | 1 (3%) | 1 | 0 | 1 (3%) | 0 | 1 | 0 |
| Dyspepsia | 1 (3%) | 1 | 0 | 1 (3%) | 1 | 0 | 0 |
| Alopecia | 1 (3%) | 0 | 1 | 0 (0%) | 0 | 0 | 0 |
| Cold sensitivity | 1 (3%) | 0 | 1 | 0 (0%) | 0 | 0 | 0 |
| Gastric hemorrhage | 1 (3%) | 0 | 1 | 0 (0%) | 0 | 0 | 0 |
| Hypoglycemia | 1 (3%) | 0 | 1 | 0 (0%) | 0 | 0 | 0 |
| Pancreatitis | 1 (3%) | 0 | 1 | 0 (0%) | 0 | 0 | 0 |
| Peripheral sensory neuropathy | 1 (3%) | 0 | 1 | 0 (0%) | 0 | 0 | 0 |
| Ascites | 1 (3%) | 1 | 0 | 0 (0%) | 0 | 0 | 0 |
| Confusion | 1 (3%) | 1 | 0 | 0 (0%) | 0 | 0 | 0 |
| Conjunctivitis | 1 (3%) | 1 | 0 | 0 (0%) | 0 | 0 | 0 |
| Creatinine increased | 1 (3%) | 1 | 0 | 0 (0%) | 0 | 0 | 0 |
| Decreased urination | 1 (3%) | 1 | 0 | 0 (0%) | 0 | 0 | 0 |
| Dysgeusia | 1 (3%) | 1 | 0 | 0 (0%) | 0 | 0 | 0 |
| Dysuria | 1 (3%) | 1 | 0 | 0 (0%) | 0 | 0 | 0 |
| Eye disorder – stye | 1 (3%) | 1 | 0 | 0 (0%) | 0 | 0 | 0 |
| Eye pain | 1 (3%) | 1 | 0 | 0 (0%) | 0 | 0 | 0 |
| Facial muscle weakness | 1 (3%) | 1 | 0 | 0 (0%) | 0 | 0 | 0 |
| Fall | 1 (3%) | 1 | 0 | 0 (0%) | 0 | 0 | 0 |
| Flushing | 1 (3%) | 1 | 0 | 0 (0%) | 0 | 0 | 0 |
| Fracture | 1 (3%) | 1 | 0 | 0 (0%) | 0 | 0 | 0 |
| Gastroesophageal reflux disease | 1 (3%) | 1 | 0 | 0 (0%) | 0 | 0 | 0 |
| Heat and cold intolerance | 1 (3%) | 1 | 0 | 0 (0%) | 0 | 0 | 0 |
| Hematuria | 1 (3%) | 1 | 0 | 0 (0%) | 0 | 0 | 0 |
| Hyponatremia | 1 (3%) | 1 | 0 | 0 (0%) | 0 | 0 | 0 |
| Insomnia | 1 (3%) | 1 | 0 | 0 (0%) | 0 | 0 | 0 |
| Localized edema | 1 (3%) | 1 | 0 | 0 (0%) | 0 | 0 | 0 |
| Nail discoloration | 1 (3%) | 1 | 0 | 0 (0%) | 0 | 0 | 0 |
| Non-cardiac chest pain | 1 (3%) | 1 | 0 | 0 (0%) | 0 | 0 | 0 |
| Periodontal disease | 1 (3%) | 1 | 0 | 0 (0%) | 0 | 0 | 0 |
| Tinnitus | 1 (3%) | 1 | 0 | 0 (0%) | 0 | 0 | 0 |
| Wheezing | 1 (3%) | 1 | 0 | 0 (0%) | 0 | 0 | 0 |
| Wound dehiscence | 1 (3%) | 1 | 0 | 0 (0%) | 0 | 0 | 0 |
| Lymphocyte count decreased | 0 (0%) | 0 | 0 | 1 (3%) | 0 | 1 | 0 |
| Acute kidney injury | 0 (0%) | 0 | 0 | 1 (3%) | 1 | 0 | 0 |
| Anemia | 0 (0%) | 0 | 0 | 1 (3%) | 1 | 0 | 0 |
| Constipation | 0 (0%) | 0 | 0 | 1 (3%) | 1 | 0 | 0 |
| Hyperbilirubinemia | 0 (0%) | 0 | 0 | 1 (3%) | 1 | 0 | 0 |
